# Supplementary material for: Mutations in PCYT2 disrupt etherlipid biosynthesis and cause a complex hereditary spastic paraplegia
Source: Brain. 2019 Oct 22;142(11):3382–97. doi: 10.1093/brain/awz291 (PMC6821184; doi:10.1093/brain/awz291)
Supplement: awz291_Supplementary_Data [file awz291_supplementary_data.zip › awz291-Suppl_data/Supplementary_Data4.zip!/Supplementary information 3 - fibroblast lipidomics.zip!/Fibroblast lipidomics/Patient1 vs Patient2/Statistical plots and data/PS species/Random forest.pdf]

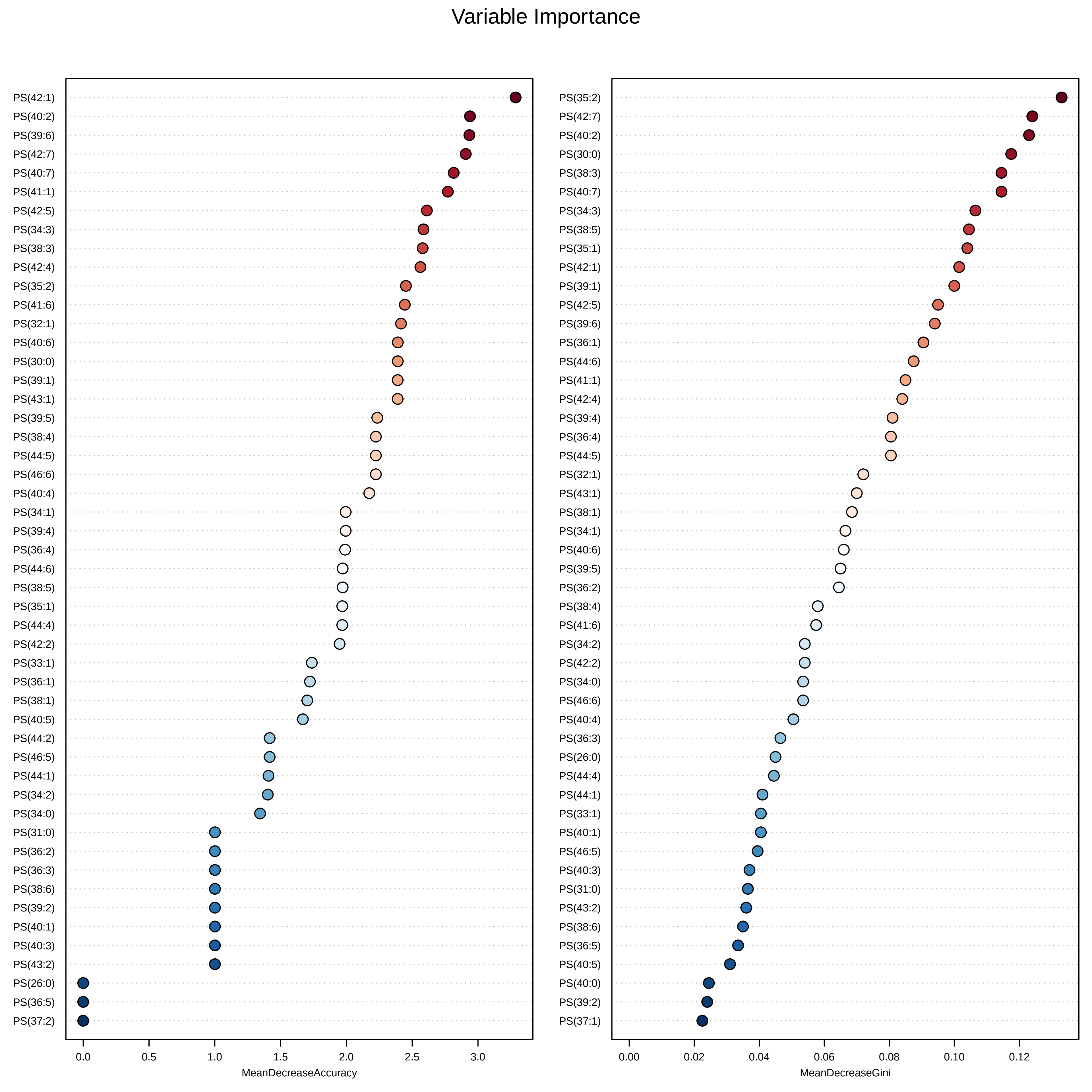

Variable Importance
PS(42:1)
PS(40:2)
PS(39:6)
PS(42:7)
PS(40:7)
PS(41:1)
PS(42:5)
PS(34:3)
PS(38:3)
PS(42:4)
PS(35:2)
PS(41:6)
PS(32:1)
PS(40:6)
PS(30:0)
PS(39:1)
PS(43:1)
PS(39:5)
PS(38:4)
PS(44:5)
PS(46:6)
PS(40:4)
PS(34:1)
PS(39:4)
PS(36:4)
PS(44:6)
PS(38:5)
PS(35:1)
PS(44:4)
PS(42:2)
PS(33:1)
PS(36:1)
PS(38:1)
PS(40:5)
PS(44:2)
PS(46:5)
PS(44:1)
PS(34:2)
PS(34:0)
PS(31:0)
PS(36:2)
PS(36:3)
PS(38:6)
PS(39:2)
PS(40:1)
PS(40:3)
PS(43:2)
PS(26:0)
PS(36:5)
PS(37:2)
0.0
0.5
1.0
1.5
2.0
2.5
3.0
MeanDecreaseAccuracy
PS(35:2)
PS(42:7)
PS(40:2)
PS(30:0)
PS(38:3)
PS(40:7)
PS(34:3)
PS(38:5)
PS(35:1)
PS(42:1)
PS(39:1)
PS(42:5)
PS(39:6)
PS(36:1)
PS(44:6)
PS(41:1)
PS(42:4)
PS(39:4)
PS(36:4)
PS(44:5)
PS(32:1)
PS(43:1)
PS(38:1)
PS(34:1)
PS(40:6)
PS(39:5)
PS(36:2)
PS(38:4)
PS(41:6)
PS(34:2)
PS(42:2)
PS(34:0)
PS(46:6)
PS(40:4)
PS(36:3)
PS(26:0)
PS(44:4)
PS(44:1)
PS(33:1)
PS(40:1)
PS(46:5)
PS(40:3)
PS(31:0)
PS(43:2)
PS(38:6)
PS(36:5)
PS(40:5)
PS(40:0)
PS(39:2)
PS(37:1)
0.00
0.02
0.04
0.06
0.08
0.10
0.12
MeanDecreaseGini
